# Efficacy of Inertial Measurement Units in the Evaluation of Trunk and Hand Kinematics in Baseball Hitting

**DOI:** 10.3390/s20247331

**Published:** 2020-12-20

**Authors:** Niroshan G. Punchihewa, Shigeaki Miyazaki, Etsuo Chosa, Go Yamako

**Affiliations:** 1 Department of Materials and Informatics, Interdisciplinary Graduate School of Agriculture and Engineering, University of Miyazaki, Miyazaki 889-2192, Japan; jb17007@student.miyazaki-u.ac.jp; 2 Department of Orthopaedics, Faculty of Medicine, University of Miyazaki, Miyazaki 889-1692, Japan; 03-5-23@med.miyazaki-u.ac.jp (S.M.); chosa@med.miyazaki-u.ac.jp (E.C.); 3 Department of Mechanical Design Systems Engineering, Faculty of Engineering, University of Miyazaki, Miyazaki 889-2192, Japan

**Keywords:** baseball batting, inertial sensors, trunk kinematics, motion analysis, reliability, accuracy, coordination

## Abstract

Baseball hitting is a highly dynamic activity, and advanced methods are required to accurately obtain biomechanical data. Inertial measurement units (IMUs) can capture the motion of body segments at high sampling rates both indoor and outdoor. The bat rotates around the longitudinal axis of the body; thus, trunk motion plays a key role in baseball hitting. Segmental coordination is important in transferring power to a moving ball and, therefore, useful in evaluating swing kinematics. The current study aimed to investigate the validity and reliability of IMUs with a sampling rate of 1000 Hz attached on the pelvis, thorax, and hand in assessing trunk and hand motion during baseball hitting. Results obtained using the IMU and optical motion capture system (OMCS) were compared. Angular displacements of the trunk segments and spine joint had a root mean square error of <5°. The mean absolute error of the angular velocities was ≤5%. The intra-class correlation coefficient (>0.950) had excellent reliability for trunk kinematics along the longitudinal-axis. Hand velocities at peak and impact corresponded to the values determined using the OMCS. In conclusion, IMUs with high sampling rates are effective in evaluating trunk and hand movement coordination during hitting motion.

## 1. Introduction

Baseball is primarily a battle between a pitcher who throws a ball at about 145 kmh^−1^ and a hitter who attempts to hit the ball pitched from 18.4 m away within less than a half of a second. Thus, baseball hitting is considered one of the most difficult sport activities [1]. Baseball hitters need a high swing speed to hit a ball long distance. The rotational motion of the body in the longitudinal axis is important in increasing swing speed [2]. This rotational motion is achieved by the sequential recruitment of muscles from the lower to the upper body segments [3]. Torso strength training can significantly increase swing speed [4]. However, the incidence of abdominal muscle injuries is increasing among professional baseball players. About 56% of these injuries were attributed to baseball hitting, and most were found in the internal/external oblique muscles [5]. Abdominal oblique muscles stabilize trunk axial rotation. The maximum muscle activity of oblique muscles can reach >100% during swing phase and follow through [6]. Peak lumbar spine rotation may contribute to lumbar disk herniation [7]. Thus, to reduce the risk of abdominal muscle and spine injuries, hitting kinematics should be considered when assigning appropriate strength and conditioning exercises.

To analyze biomechanics in human movements, the use of a wireless inertial measurement unit (IMU), which is a miniature sensor unit comprising an accelerometer, gyroscope, and, occasionally, a magnetometer, has been increasing [8]. We can easily attach a light weight IMU on human body segments and can calculate kinematic parameters such as body–segmental orientation and joint angles between adjacent segments. The validity and reliability of IMUs for gait have been assessed in previous studies [9,10]. However, the applicability of IMUs in the analysis of trunk motion in fast moving activities including baseball hitting remains unclear. Hence, the current study aimed to evaluate the validity and reliability of IMUs in analyzing trunk kinematics during baseball hitting. We hypothesized that IMUs with a sampling rate of 1000 Hz can be used in assessing hitting coordination.

## 2. Materials and Methods

### 2.1. Participants

Eight right-handed male baseball players (age: 19.9 ± 1.4 years; height: 1.7 ± 0.1 m; weight: 69.0 ± 10.9 kg) who are part of the Miyazaki University Baseball Club voluntarily participated in the current study. All participants had no known history of musculoskeletal or neurological diseases. The study protocol was approved by the ethics committee of the university, and a written informed consent was obtained from each participant prior to data collection.

### 2.2. Instrumental Setup

The hitting motion data captured using three IMUs (sampling rate of 1000 Hz, SS-MS-HMA200G60 (accelerometer (200 G, G = 9.81 ms^−2^), gyroscope (6000 °s^−1^), magnetometer (10 gauss)), size: 36 mm (width) × 53 mm (length) × 11 mm (depth), weight: 32 g, Sports Sensing Co., Ltd., Fukuoka, Japan). Each IMU has an internal memory that can store raw sensor data. All IMUs were synchronized using a wireless transceiver. Fully charged IMUs were kept for 10 min after switching on to allow the sensors achieve a steady state temperature.

Accelerometer and magnetometer were calibrated before hitting measurement. Accelerometer data were collected while keeping IMUs at rest on a flat surface in six different orientations. The accelerometer was calibrated by zeroing-out the offset [11]. Magnetometer data were recorded while IMUs were randomly rotated for 45 s inside the target volume of data capture. The Magneto 1.2 software was used to calibrate the magnetometer [12]. This software has adopted the ellipsoid fitting technique to compensate for soft and hard iron distortions [13]. The magnetic field is important in obtaining orientation data from the IMU. However, it is usually distorted due to construction materials and other electronic equipment inside motion laboratories. Thus, IMU data should be acquired within 30 s in a single trial, thereby keeping IMUs over 1 m away from ground level [14]. In this study, we followed the guidelines during data collection.

IMUs were used for detecting segment motion in the thorax, pelvis, and hand. One IMU was attached between the manubrium and xiphoid process of the sternum. Another IMU was attached between the left and right posterior iliac spines. Both IMUs were attached such that the *x*-axis corresponded to the medio-lateral direction and the *y*-axis to the longitudinal axis based on anatomical landmarks. The third IMU was attached on the dorsal side of the leading hand over the batting glove using a double-sided tape and was secured with an elasticized bandage. The positive *y*-axis was aligned with the long axis toward the proximal direction of the hand.

### 2.3. Data Collection during Hitting Motion

After performing self-selected warm-up exercises, players hit a baseball over a tee-pole for 10 times with an interval of not >30 s between each swing. Players could adjust the tee-pole to their preferred height to perform bat swing. A safety net was used to trap the batted balls. Before hitting measurement, players were instructed to stand still for 5 s while facing the home plate, and the posture was recorded to determine the initial position (static pose). After all the trials were completed, IMU data were transferred to a computer.

### 2.4. Calculation of Kinematic Parameters

Kinematic parameters including angular displacements and velocities of the thorax, pelvis, and spine were calculated using IMU data with a script written in MATLAB (version R2017b, MathWorks^®^, Natick, MA, USA). The angular rates of gyroscope data at static position were averaged and subtracted from each axis to remove initial bias. The acceleration and angular rates of each IMU were filtered using a fourth-order, low-pass Butterworth filter (cutoff frequency of 20 Hz). Orientations of the thorax, pelvis, and hand were calculated with IMU data using gradient-descent fusion algorithm and were expressed as unit quaternion (*q*(*t*)) with respect to a common global coordinate system defined by gravity vector from the accelerometer and earth’s magnetic field from the magnetometer [15]. Derived orientation is a result of the minimization of predicted and estimated IMU values using a two-stage gradient-descent optimization algorithm. This open-source fusion algorithm is computationally efficient and has been used as a benchmark to evaluate other orientation estimation algorithms, thus used in our study [16,17].

**Figure 1:**
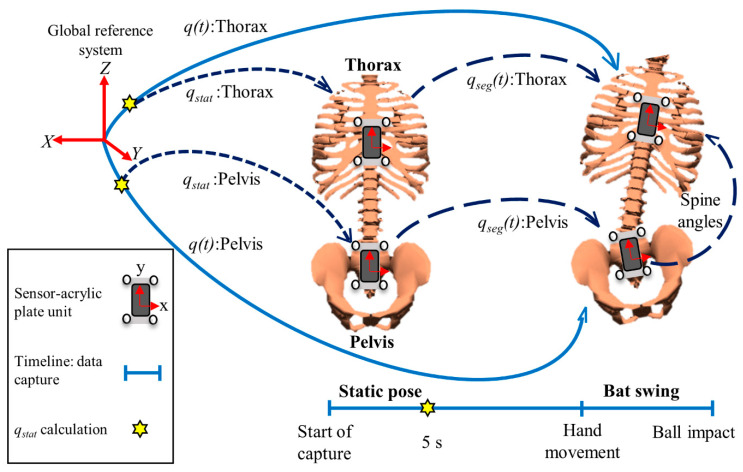
Orientation definition in quaternion for both static position and hitting a baseball off a tee-pole.

Static quaternion (*q_stat_*) in thorax and pelvis were calculated within the last 0.5 s in static pose (Figure 1). Segment orientation (*q_seg_*(*t*)) during swing motion was calculated with respect to *q_stat_* using Equation (1):

(1) $$q_{seg}\left(t\right)=q\left(t\right)⨂q^{*}{}_{stat}$$

where q* represents quaternion conjugate and ⨂ represents quaternion multiplication. *q_seg_*(*t*) represents the quaternion of each segment. The y-axis had the highest range of motion in the thorax and pelvis during baseball swing, which corresponded to the longitudinal axis of each segment. Thus, *q_seg_*(*t*) was converted to Euler angles using the YXZ Euler sequence to prevent gimbal lock [18,19].

Spine angles were calculated from the thorax and pelvis orientations using the joint coordinate system recommended by the international society of biomechanics [20,21]. The *x*-axis of the pelvis was considered as the flexion axis, whereas the *y*-axis of the thorax was considered as the axial rotation of the spine. The antero-posterior axis, referred to as the floating axis, was calculated by taking the cross product of the abovementioned axes for spine lateral flexion [22]. The angular velocity of each segment could be directly acquired using calibrated gyroscope data. Spine angular velocity was calculated as the first derivative of the time-dependent spine angular displacements.

### 2.5. Estimation of Hitting Events and Hand Motion

**Figure 2:**
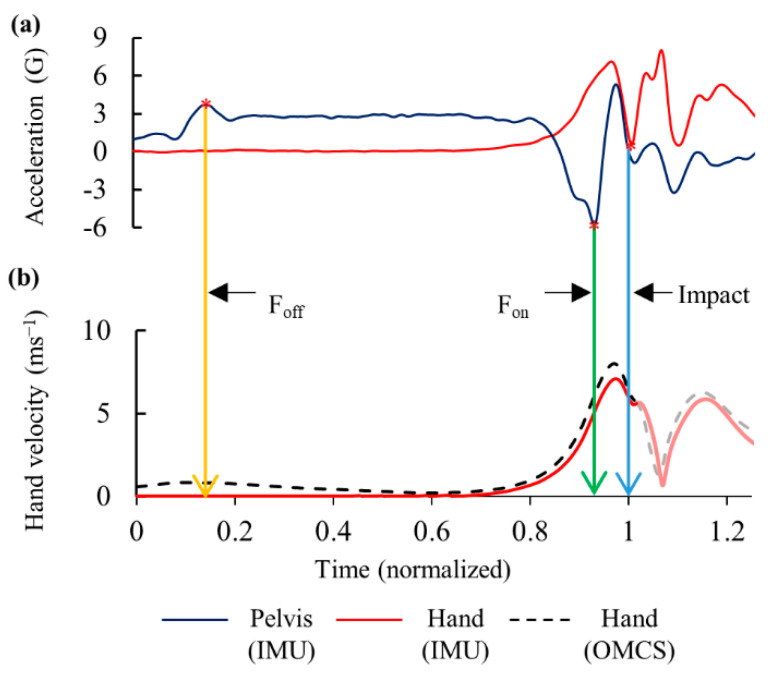
Typical graph of (**a**) Foot off (F_off_), foot on (F_on_) and impact events detected by inertial measurement units (IMUs) and (**b**) linear velocity of the hand determined by IMU and optical motion capture system (OMCS).

To describe the timing of baseball swing in tee batting, foot-off and foot-on events were detected using pelvis acceleration data (Figure 2a). Foot events and impact time from the IMUs were calculated according to a method described previously [23].

Acceleration data of the hand IMU were used to detect impact time and to calculate linear velocity. A negative acceleration peak value approximately opposite to the direction of impact (*y*-axis) was used to detect impact point [23]. Gravity vector was removed by transforming acceleration data into a global reference system and was integrated to calculate resultant velocity vector (Figure 2b). A threshold value of 0.1 G was set to recognize initial hand movement during the forward swing phase.

### 2.6. Kinematic Data Validation with the OMCS

**Figure 3:**
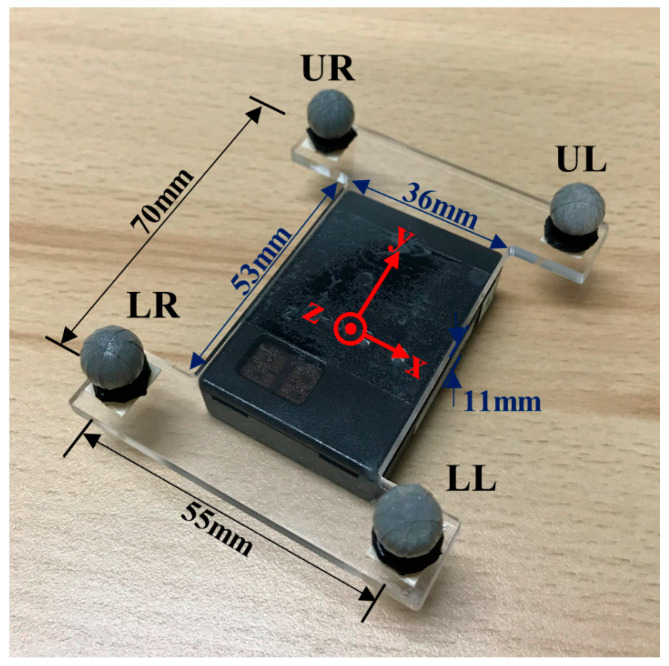
Acrylic plate attached on each IMU on the trunk segments. UR: upper right, UL: upper left, LR: lower right, LL: lower left.

Kinematic data measured using IMUs and the OMCS were compared (sampling rate of 250 Hz, 13 cameras, VICON Motion Systems Ltd., Oxford, UK). Cameras were calibrated before the data collection using an active wand. All cameras provided residuals of the image and world errors less than 0.2 mm. A 5 V pulse trigger was used to synchronize both IMUs and the OMCS. To validate the measurement accuracy of IMUs, an I-shaped acrylic plate (thickness: 3 mm, weight: 14 g) with four reflective markers (10 mm in diameter) was attached on the IMUs (Figure 3) placed on the thorax and pelvis. Both plates with IMU were further secured with an elasticized bandage. These marker position data measured using the OMCS were used to construct the local coordinate systems of each body segment. The local coordinate system corresponded to that of the IMU.

A reflective marker was attached on top of the IMU attached on the hand to assess hand motion. Another marker was attached on the baseball to identify bat–ball impact.

Marker trajectory data during hitting were filtered using fourth-order low-pass Butterworth filter (cutoff frequency of 20 Hz). The local coordinate system of each segment was calculated using Equations (2)–(4):

(2) $$X=\frac{\left(\left[LL+UL\right]÷2-\left[LR+UR\right]÷2\right)}{\left\|(\left[LL+UL\right]÷2-\left[LR+UR\right]÷2)\right\|}$$

(3) $$Z=X\times \frac{\left(\left[UR+UL\right]÷2-\left[LR+LL\right]÷2\right)}{\left\|(\left[UR+UL\right]÷2-\left[LR+LL\right]÷2)\right\|}$$

(4) Y=Z×X

where *LL* is the lower left marker, *LR* is the lower right marker, *UL* is the upper left marker, and *UR* is the upper right marker (Figure 3). The [*X, Y, Z*] rotation matrix was converted into a unit quaternion to calculate segmental angular displacements. Segmental angular velocity (ω) was calculated using Equations (5) and (6):

(5) $${\dot{q}}_{seg}=\frac{q_{seg}\left(t+1\right)-q_{seg}\left(t-1\right)}{2\Delta t}$$

(6) $$\omega^{\prime }=2\left(q^{*}{}_{seg}\left(t\right)⨂{\dot{q}}_{seg}\right)$$

where ∆*t* is the time interval between two data points (4 ms). Since ω’ = [ω_0_, ω_1_, ω_2_, ω_3_] represents the quaternion format, ω was derived as [ω_1_, ω_2_, ω_3_] [24]. A fifth-order median filter was used to reduce noise in the angular velocity data. The median filter was used in both systems for consistency.

Position data of the hand marker were used to derive the hand velocity. The first frame prior to the displacement of marker attached on the ball was visually identified as bat–ball impact [25].

### 2.7. Statistical Analysis

The swing duration was defined as time when the hand started moving backward before swinging to bat–ball impact detected using the OMCS. One trial was excluded from the data analysis due to IMU data loss during data capture. In total, 79 trials were used for the analysis.

Angular displacements and velocities of the thorax, pelvis, and spine were the trunk kinematics that should be evaluated. The root mean square error (RMSE) across the angular displacement curves between the IMUs and OMCS was calculated, and the error was averaged to estimate for accuracy. An RMSE of <5° was considered excellent and between 5° and 10° as good [26]. The accuracy of the angular velocity curves was measured using the mean absolute error (MAE), which was represented as a percentage of the peak angular velocity that occurred before bat–ball impact.

To evaluate for reliability, intra-class correlation coefficient (ICC; a two-way mixed model for absolute agreement) was calculated using the Statistical Package for the Social Sciences software (version 22.0, IBM Corp., Tokyo, Japan). The peak values of the trunk angular displacements were obtained after bat–ball impact (mainly in the *y*-axis; axial rotation). Thus, angular displacement at impact was set as the reliability measurement. The peak values were used to evaluate the reliability of angular velocities. An ICC of <0.5 was considered poor; between 0.5 and 0.75, moderate; between 0.75 and 0.9, good; and >0.9, excellent [27]. Bland–Altman analysis was performed to understand the agreement between two measurement systems [28]. The upper and lower limits were calculated as mean bias ±1.96 times the standard deviation (SD). Moreover, the peak hand velocity and impact time differences between the measurement systems were compared.

## 3. Results

### 3.1. Swing Description Using IMUs

**Figure 4:**
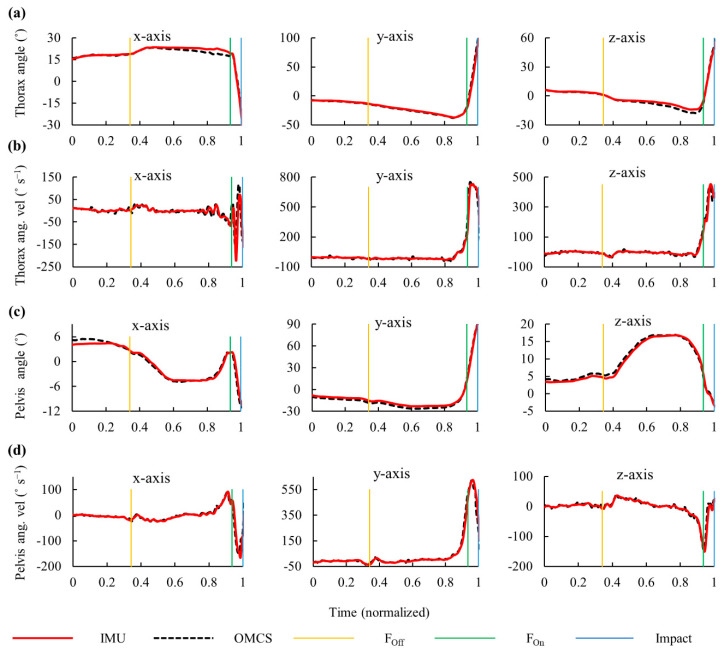
Typical graph of time-dependent change in angular displacement and angular velocity (ang. vel.) of the thorax ((**a**,**b**)) and pelvis ((**c**,**d**)) segments measured using the inertial measurement units (IMUs) and the optical motion capture system (OMCS). Vertical lines represent foot-off (F_off_), foot-on (F_on_), and impact events.

**Figure 5:**
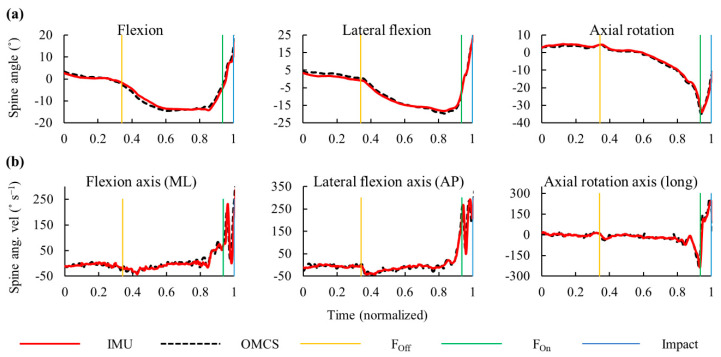
Typical graph of time-dependent change in (**a**) angular displacement and (**b**) angular velocity (ang. vel.) of the spine joint measured using the inertial measurement units (IMUs) and the optical motion capture system (OMCS). The vertical lines represent foot-off (F_off_), foot-on (F_on_), and impact events. ML: Medio-lateral axis, AP: Antero-posterior axis, Long: Longitudinal axis.

**Figure 6:**
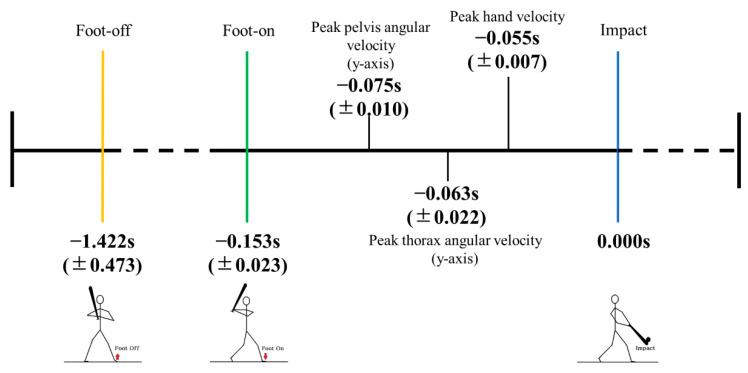
Mean timing (standard deviation) of segmental peak velocities and key events during baseball swing.

After the front foot was lifted off the ground, the thorax was rotated along the *y*-axis clockwise (Figure 4a), thereby increasing the spine axial rotation in the negative direction (Figure 5a). The pelvis angular velocity increased rapidly followed by the thorax just before the front foot was planted on the ground. The maximum angular velocities along the longitudinal axis for the thorax, pelvis, and spine were observed before the impact (Figure 4 and Figure 5). The hand velocity maximized after the trunk segmental velocities reached its peak. The average time (SD) from foot-off to impact was 1.422 (0.473) s, and that from foot-on to impact was 0.153 (0.023) s (Figure 6).

### 3.2. Validity and Reliability of IMUs

IMUs had excellent validity in measuring hitting kinematics. For angular displacement, the RMSEs were below 5° in the thorax, pelvis, and spine (Table 1). For angular velocity, the MAEs were almost ≤5% in the trunk segments and spine joint. At impact, an excellent reliability was observed in the angular displacements except for the medio-lateral axis of the spine (Table 2). The peak angular velocities had excellent reliability with an ICC of >0.950.

Bland–Altman analysis showed good consistency in the angular displacements at impact with a mean bias of around ±2.5°, and the limit of agreement was within ±10° except the medio-lateral axis of the spine (Table 2).

The hand velocity at peak and impact were 7.94 (1.14) ms^−1^ and 4.70 (0.83) ms^−1^, respectively. The errors were 0.63 (0.34) ms^−1^ for peak (mean absolute error percentage (MAEP): 7.18% and ICC: 0.920) and 0.31 (0.40) ms^−1^ for impact (MAEP: 8.68%, ICC: 0.905). The impact time detected using the hand IMU was extremely similar to that identified using the OMCS. The mean error (SD) was 0.007 (0.004) s, the RMSE was 0.008 s, and the reliability was excellent (ICC: 1.000).

## 4. Discussion

Three wireless IMUs with a sampling rate of 1000 Hz could detect the characteristics of hitting motion. The different timing of peak velocity for the pelvis, thorax, and hand measured using the IMUs indicated the sequential motion of proximal to distal segments [29]. The time difference in peak velocity between the thorax and hand as well as the pelvis and thorax were 8 and 12 ms, respectively (Figure 6). This duration corresponds to 2–3 data points when the sampling frequency is set at 250 Hz. Further, timing of the bat ball impact is heavily affected when filtering the trajectory data that are captured at lower sampling rates [25]. It has been advised to use unfiltered data to calculate bat head speed and to increase sampling rate [30]. Thus, in this study, the sampling frequency of IMUs was set to 1000 Hz to increase data points. Previous studies have shown that the peak angular velocities of the thorax and pelvis lie between 857–937 °s^−1^ and 678–897 °s^−1^ in adult hitters [30,31,32]. These results are consistent with those of the current study (thorax: 973 °s^−1^, pelvis: 643 °s^−1^). Small discrepancies can be expected due to different experimental settings and the skill levels of the participants. Changes in rotational velocity within a short period may contribute to not only hitting a ball at long distance but inducing spine and muscle injuries. Thus, trunk motion should be monitored accurately to improve batting performance and prevent injuries.

The hand acceleration profile obtained using the IMU was used to detect impact time based on our previous study [23]. In this study, the accuracy of impact detection was 8 ms, with an excellent reliability (ICC: 1.000). The hands are continuously accelerated by increasing elbow extension velocities according to impact, and the acceleration reaches its peak toward the direction of impact.

Consistency in measurement data is important in evaluating swing kinematics in individual players to improve hitting performance. In the Bland–Altman analysis, the IMUs, compared with the OMCS, had good consistency. OMCSs are the gold standard for the kinematic analysis of baseball hitting in a fixed laboratory setup [31,33]. While marker displacement data can be captured <0.2 mm errors in a well calibrated setup, there are practical difficulties such as blind spots to detect markers and to obtain accurate higher derivative motion data such as velocity and acceleration. However, the use of IMUs is advantageous. That is, complex movements can be captured without considering data loss due to marker occlusion in the cameras of the OMCSs. Further, angular velocity and acceleration data are directly available, and they are useful in calculating joint movements and forces using inverse dynamic techniques [34].

The spine flexion had moderate reliability at impact. However, the spine axial rotation, which is the primary motion of the baseball hitting, had excellent reliability. ICC is directly influenced by the inter-subject variability; thus, the values could be lower in associated movements than in the primary motion [35].

The current study had several limitations that should be addressed. First, the comparison between IMUs and the OMCS was confined to laboratory settings; thus, tee-pole batting was performed instead of hitting a pitched ball. This measurement environment might have affected hitting kinematics. Second, the sampling rate of the OMCS was set at 250 Hz. This rate would not be sufficient in detecting peak velocities because these values were derived based on the time derivative of the marker displacement. Third, although the effect of magnetic distortion for orientation estimation could not be eliminated completely, the error magnitude was not quantified. Fourth, we could not evaluate the accuracy of the IMU after impact due to the marker occlusion of the OMCS. The upper extremities were closer to the body after impact, and the markers of the thorax were hidden to the cameras. Nevertheless, we believe that the validity of IMU measurements did not change after bat–ball impact since the peak velocity, which occurred before impact, had excellent validity. Finally, the weight of the acrylic plate might have increased the relative motion between the IMU and the body segment, which resulted in skin artifact.

## 5. Conclusions

IMU could accurately assess trunk and hand kinematics when hitting a baseball off a tee-pole. Its validity and reliability in evaluating the angle and velocity of the trunk and hand were good to excellent. A high sampling frequency was required for detecting time difference between the kinematic peaks of each segment and for evaluating coordination in hitting motion.

## Figures and Tables

**Table 1:** Root mean square error (RMSE) in degrees and the mean absolute error (MAE) reported as a percentage of the peak values of the respective trunk kinematics.

Segment/Joint	Axis	RMSE (°) (Angular Displacement)	MAE (%) (Angular Velocity)
Thorax	x	2.16	5.06
	y	3.78	3.66
	z	2.64	2.37
Pelvis	x	1.57	5.58
	y	1.94	1.59
	z	1.36	2.13
Spine	Flexion (ML) ^1^	2.69	4.14
	Lateral flexion (AP) ^2^	1.83	1.37
	Axial rotation (long) ^3^	1.49	4.48

^1^ ML: Medio-lateral axis, ^2^ AP: Antero-posterior axis, ^3^ long: Longitudinal axis.

**Table 2:** Mean (standard deviation) measurements of IMUs, mean bias with upper and lower bound of the limit of agreement (LOA [UB, LB]), and intra-class correlation coefficient (ICC) between measurement systems at impact in angular displacement (in degrees except ICC) and at peak in angular velocity (in degrees per second except ICC) of the thorax, pelvis, and spine.

		Angular Displacement at the Impact (°)				Peak Angular Velocity (°s^−1^)			
Segment/Joint	Axis	Mean (SD)	Bias	LOA (UB, LB)	ICC	Mean (SD)	Bias	LOA (UB, LB)	ICC
Thorax	x	−21.2 (4.8)	−1.87	(2.18, −5.93)	0.909	237.5 (156.8)	25.02	(129.42, −79.38)	0.955
	y	88.6 (11.5)	−1.06	(6.55, −8.67)	0.969	973.9 (248.0)	−0.21	(100.20, −100.62)	0.988
	z	40.5 (9.3)	2.22	(5.81, −1.36)	0.951	423.9 (117.9)	10.5	(68.18, −47.18)	0.981
Pelvis	x	−1.0 (6.2)	−2.53	(0.42, −5.48)	0.951	145.5 (49.5)	8.01	(20.73, −4.72)	0.989
	y	79.2 (13.2)	−0.63	(3.26, −4.53)	0.994	643.0 (49.7)	6.34	(19.23, −6.54)	0.991
	z	−13.7 (8.2)	−1.54	(1.56, −4.64)	0.98	423.9 (117.9)	2.62	(9.51, −4.28)	0.998
Spine	Flexion (ML) ^1^	0.0 (7.4)	−8.66	(−0.01, −17.30)	0.632	346.5 (172.5)	8.76	(101.45, −83.93)	0.982
	Lateral flexion (AP) ^2^	29.8 (7.6)	1.42	(6.81, −3.97)	0.953	339.7 (110.3)	25.45	(91.64, −40.73)	1.37
	Axial rotation (long) ^3^	2.3 (8.2)	−1.08	(1.60, −3.76)	0.988	705.5 (410.6)	43.9	(185.17, −97.37)	4.48

^1^ ML: Medio-lateral axis, ^2^ AP: Antero-posterior axis, ^3^ long: Longitudinal axis.
